# Restoration of E-cadherin expression by selective Cox-2 inhibition and the clinical relevance of the epithelial-to-mesenchymal transition in head and neck squamous cell carcinoma

**DOI:** 10.1186/1756-9966-33-40

**Published:** 2014-05-10

**Authors:** Ryoichi Fujii, Yorihisa Imanishi, Katsushi Shibata, Nobuya Sakai, Koji Sakamoto, Seiji Shigetomi, Noboru Habu, Kuninori Otsuka, Yoichiro Sato, Yoshihiro Watanabe, Hiroyuki Ozawa, Toshiki Tomita, Kaori Kameyama, Masato Fujii, Kaoru Ogawa

**Affiliations:** 1 Department of Otorhinolaryngology-Head and Neck Surgery, Keio University School of Medicine, 35 Shinanomachi, Shinjuku, Tokyo 160-8582, Japan; 2 Department of Functional Genomics, Faculty of Pharmaceutical Sciences, Himeji Dokkyo University, Himeji, Japan; 3 Department of Otorhinolaryngology, Saiseikai Utsunomiya Hospital, Utsunomiya, Japan; 4 Department of Otorhinolaryngology, Kawasaki Municipal Hospital, Kawasaki, Japan; 5 Department of Otorhinolaryngology-Head and Neck Surgery, Keiyu Hospital, Yokohama, Japan; 6 Department of Pathology, Keio University School of Medicine, Tokyo, Japan; 7 National Institute of Sensory Organs, National Tokyo Medical Center, Tokyo, Japan

**Keywords:** E-cadherin, Cox-2 inhibition, Head and neck squamous cell carcinoma (HNSCC), Epithelial-to-mesenchymal transition (EMT), Lymph node metastasis

## Abstract

**Background:**

The epithelial-to-mesenchymal transition (EMT) accompanied by the downregulation of E-cadherin has been thought to promote metastasis. Cyclooxygenase-2 (Cox-2) is presumed to contribute to cancer progression through its multifaceted function, and recently its inverse relationship with E-cadherin was suggested. The aim of the present study was to investigate whether selective Cox-2 inhibitors restore the expression of E-cadherin in head and neck squamous cell carcinoma (HNSCC) cells, and to examine the possible correlations of the expression levels of EMT-related molecules with clinicopathological factors in HNSCC.

**Methods:**

We used quantitative real-time PCR to examine the effects of three selective Cox-2 inhibitors, i.e., celecoxib, NS-398, and SC-791 on the gene expressions of E-cadherin (CDH-1) and its transcriptional repressors (SIP1, Snail, Twist) in the human HNSCC cell lines HSC-2 and HSC-4. To evaluate the changes in E-cadherin expression on the cell surface, we used a flowcytometer and immunofluorescent staining in addition to Western blotting. We evaluated and statistically analyzed the clinicopathological factors and mRNA expressions of Cox-2, CDH-1 and its repressors in surgical specimens of 40 patients with tongue squamous cell carcinoma (TSCC).

**Results:**

The selective Cox-2 inhibitors upregulated the E-cadherin expression on the cell surface of the HNSCC cells through the downregulation of its transcriptional repressors. The extent of this effect depended on the baseline expression levels of both E-cadherin and Cox-2 in each cell line. A univariate analysis showed that higher Cox-2 mRNA expression (p = 0.037), lower CDH-1 mRNA expression (p = 0.020), and advanced T-classification (p = 0.036) were significantly correlated with lymph node metastasis in TSCC. A multivariate logistic regression revealed that lower CDH-1 mRNA expression was the independent risk factor affecting lymph node metastasis (p = 0.041).

**Conclusions:**

These findings suggest that the appropriately selective administration of certain Cox-2 inhibitors may have an anti-metastatic effect through suppression of the EMT by restoring E-cadherin expression. In addition, the downregulation of CDH-1 resulting from the EMT may be closely involved in lymph node metastasis in TSCC.

## Background

Head and neck squamous cell carcinoma (HNSCC) is the sixth most common cancer with an annual incidence of over 560,000 cases worldwide [1]. Despite various advances in combined modality therapy, the survival rate of HNSCC patients has not improved over the past two decades, due largely to the uncontrollable metastasis to lymph nodes and distant organs [2]. Cervical lymph node metastasis in particular has been considered the most important adverse prognostic factor in HNSCC [3-5]. More effective strategies based on a better understanding of the molecular mechanisms that lead to metastasis are thus indispensable.

Recent progress in tumor biology indicates that the initial steps during the sequential process of metastasis are notably analogous to the epithelial-to-mesenchymal transition (EMT) in which cells lose epithelial features including cell adhesion and gain mesenchymal traits including cell motility during embryogenesis and wound healing [6,7]. In the tumor context, the acquisition of the EMT, accompanied by functional loss of E-cadherin that maintains intercellular adhesion, stimulates the dissemination of single tumor cells from primary sites through the loss of cell-to-cell contact, thereby endowing cells with metastatic abilities [6-8]. At the transcriptional level, E-cadherin is downregulated by several transcriptional repressors including snail, slug, DeltaEF1/ZEB1, SIP1 (Smad interacting protein 1)/ZEB2, E12/E47, and twist, by binding to E-box promoter elements of CDH-1, a gene encoding human E-cadherin [6-8]. We recently reported that SIP1 expression was inversely correlated with E-cadherin expression in HNSCC cells, and that the downregulation of E-cadherin and upregulated nuclear localization of SIP1 were independently correlated with delayed neck metastasis in stage I/II tongue squamous cell carcinoma (TSCC) [9]. However, a practical therapeutic approach that leads to the suppression of the EMT has not been developed to control the progression of cancers, including HNSCC.

Cyclooxygenase-2 (Cox-2), an isoform of the Cox enzymes inducible in response to proinflammatory cytokines and growth factors, catalyzes the biosynthesis of prostanoids including prostaglandin E_2_ (PGE_2_), thereby playing important roles in the regulation of various cellular functions under physiologic and pathologic conditions including carcinogenesis [10-13]. Increased expression of Cox-2 has been found in a variety of human malignancies, including HNSCC [14-16]. Previous studies have reported several mechanisms by which Cox-2 contributes to carcinogenesis as well as cancer progression, including the activation of carcinogens [17], resistance to apoptosis [18,19], immunosuppression [20,21], the promotion of angiogenesis [11,22], the stimulation of proliferation [23] and invasiveness [24], and the autocrine activity of estrogen [25]. Such a multifaceted function of Cox-2 in conferring the malignant phenotype strongly suggested that Cox-2 is an attractive preventive and therapeutic target for various cancers [12,13,26-29]. A number of clinical trials have been carried out to examine the benefit of Cox-2 inhibitors, such as celecoxib, in the chemoprevention of premalignant lesions such as familial adenoma polyposis (FAP) [30], Barrett’s esophagus [31], and oral premalignant lesions [32], as well as in the treatment of advanced cancers in combination with chemotherapy [33-36]. However, these trials could demonstrate neither a significant chemopreventive effect nor any additional therapeutic effect of celecoxib on clinical outcomes, except in FAP, suggesting that the optimal applications of Cox-2 inhibitors should be reconsidered, and that further research is necessary regarding the various mechanisms underlying the anti-cancer effects of Cox-2 inhibitors against tumors.

An inverse relationship between E-cadherin and Cox-2 and its molecular mechanism in cancer cells was first shown in non-small cell lung cancer (NSCLC), in which Cox-2 overexpression led to decreased E-cadherin expression through the upregulation of PGE_2_ and transcriptional repressors of E-cadherin, whereas the inhibition of Cox-2 showed an inverse regulation of those molecules [37]. A similar effect of Cox-2 inhibitors that reverse the EMT by restoring E-cadherin expression was also found in subsets of colon, gastric, and bladder cancer cells [38-43]. However, in HNSCC, neither the effect of Cox-2 inhibitors on the regulation of E-cadherin expression nor its specific mechanism has been examined to date, except for a study that investigated interleukin-1β (IL-1β)-induced upregulation of Snail leading to EMT [44].

We conducted the present study to determine whether selective Cox-2 inhibitors restore the expression of E-cadherin through the downregulation of its transcriptional repressors to suppress the EMT in HNSCC cells, and to determine whether the gene expression levels of the molecules that are implicated in the EMT are correlated with clinicopathological parameters in HNSCC.

## Methods

### Cell culture

We used six cell lines established from human HNSCC: HSC-2 derived from the floor of the mouth; HSC-3, HSC-4, and SAS from the tongue; and KB and FaDu from the pharynx. The human fibrosarcoma cell line HT-1080 was used as the negative control for E-cadherin expression. The cells were maintained in Dulbecco’s modified Eagle’s medium (DMEM) (HSC-2, HSC-3, HSC-4, KB, and FaDu), or a mixture of DMEM and Ham’s F-12 (SAS), or minimal essential medium (HT-1080), supplemented with 10% fetal bovine serum (FBS) in a humidified incubator (37°C, 5% CO_2_).

### Inhibition of Cox2 using its specific inhibitors

HSC-2 and HSC-4 cells were seeded in six-well plates at a density of 2 × 10^5^ cells per well and incubated overnight in 10% FBS medium. The cells were then treated with different selective Cox-2 inhibitors: 50 μM of celecoxib (Toronto Research Chemicals, Toronto, Ontario, Canada), 80 μM of NS-398 (Cayman Chemical, Ann Arbor, MI, USA), or 20 μM of SC-791 (Calbiochem, Darmstadt, Germany). These concentrations of each Cox-2 inhibitor were found to be optimal with no toxic effect on cell viability up to 48 h based on our preliminary experiments for this purpose. Treatments with only dimethyl sulfoxide (DMSO) (Nacalai Tesque, Kyoto, Japan) used as a solvent for the inhibitors were set as the control. For the evaluation of changes in gene expression associated with Cox-2 inhibition, total RNA was extracted after a 12-h incubation.

### Quantitative real-time PCR

Total RNA from cell lines or fresh frozen tissues was isolated using Trizol reagent (Invitrogen, Carlsbad, CA) and reverse-transcribed into cDNA using random hexamer primer and SuperScript II reverse transcriptase (Invitrogen) according to the manufacturer’s instructions. Quantitative real-time polymerase chain reaction (PCR) was performed using the 7500 Fast Real-Time PCR system instrument and software (Applied Biosystems, Foster City, CA) following the manufacturer’s protocol. Specific primers and probes were obtained from Applied Biosystems as TaqMan® Gene Expression Assays, with the following IDs: human E-cadherin/*CDH-1*, Hs00170423_m1; Snail/*SNAI1*, Hs00195591_m1; SIP1/*ZFHX1B*, Hs00207691_m1; twist/*TWIST1*, Hs00361186_m1; Cox-2/*PTGS2*, Hs01573471_m1; and GAPDH (glyceraldehyde-3-phosphate dehydrogenase)/*GAPDH*, Hs99999905_m1. The PCR amplification conditions were: 20 s at 95°C followed by 40 cycles of 3 s denaturation at 95°C and 30 s annealing at 60°C. We quantified the relative expression levels of the genes by the standard curve method, and we compared the levels after normalization using those of GAPDH used as an endogenous control.

### Flowcytometric analysis

For the quantitative analysis of E-cadherin expression at protein level, we harvested cells that had been treated with each of the selective Cox-2 inhibitors for 24 h, using a cell dissociation solution (C 5914, Sigma-Aldrich, St. Louis, MO). Based on our preliminary experiments for this purpose, we identified the following optimal concentrations of each Cox-2 inhibitor: 25 μM of celecoxib, 40 μM of NS-398, and 10 μM of SC-791. After blocking incubation in 0.5% bovine serum albumin (BSA) in 1 × phosphate-buffered saline (PBS) for 10 min, we labeled the cells with PE-conjugated anti-human E-Cadherin antibody (BioLegend, San Diego, CA) for 1 h, followed by DNA staining using 7-AAD Viability Dye (Beckman Coulter, Indianapolis, IN) for 5 min. Control cells were labeled with PE-conjugated mouse IgG1, κ isotype ctrl (BioLegend). We then analyzed the E-cadherin expression on the cells using the Epics XL-MCL™ Flow Cytometer (Beckman Coulter). Data are presented as the median, mean, and mode of fluorescence intensity of the cells counted.

### Western blotting

The cells treated under the same conditions as those for flowcytometry were lysed in lysis buffer (50 mM Tris pH 7.5, 5 mM EDTA, 150 mM NaCl, 1% Triton-X100) containing 1 mM PMSF, 10 μg/ml leupeptin, 1 μg/ml pepstatin, 1 mU/ml aprotinin, 50 mM sodium fluoride, 2 mM sodium orthovanadate, and 50 nM Calculin A (Cell signaling). The protein concentration in the cell lysates was determined by the Bradford protein assay (Bio-Rad). Twenty μg of total proteins were separated by SDS-PAGE and transferred to polyvinylidene difluoride membranes (Amersham). The membranes were blocked with 5% skim milk in PBS containing 0.1% Tween 20, and probed with mouse anti-E-cadherin antibody (BD Biosciences) at 1:1000 dilution overnight at 4°C. Subsequently, the membranes were incubated with horseradish peroxidase-conjugated anti-mouse IgG sheep antibody (Amersham) for 1 h. The reactive proteins were visualized using ECL-plus (Amersham) according to the manufacturer’s instructions. Equal loading of proteins was confirmed by probing the membranes with mouse anti- β-actin antibody (Sigma).

### Immunofluorescent staining

HSC-2 cells for immunofluorescent staining of E-cadherin were seeded in slide chambers (IWAKI, Tokyo, Japan) and treated with 25 μM of celecoxib or DMSO for 24 h. After washing the cells extensively with PBS, we fixed the cells with cold methanol for 10 min at -20°C. After washing with PBS, the cells were incubated with Alexa Fluor 488-conjugated anti-E-cadherin antibody (Santa Cruz Biotechnology, Dallas, TX) at 1:200 dilution in PBS for 1 h. The nuclei were visualized by staining with Hoechst 33258 (Sigma-Aldrich). Stained cells were then mounted with Prolong Gold Antifade Reagent (Invitrogen). The fluorescent images were captured through a fluorescence microscope (Olympus, Tokyo, Japan).

### Patients and tissue samples

Human tissue specimens were obtained from patients with histologically verified tongue squamous cell carcinoma (TSCC) who underwent primary surgery at the Department of Otorhinolaryngology–Head and Neck Surgery, Keio University Hospital (Tokyo, Japan) between 2003 and 2011. Informed consent from patients and approval from our Institutional Ethics Review Board were obtained for the use of the clinical materials in the present study. Materials from patients who had received chemotherapy or radiotherapy prior to surgery or who previously had double cancer in the head and neck region were excluded from the study.

In addition to formalin fixation for routine histopathological diagnosis, fresh tumor tissues and, when possible, noncancerous mucosal tissues distant from the TSCC lesion were collected immediately after resection, placed separately in an RNA stabilization regent (RNAlater, Qiagen, Valencia, CA), and stored at −80°C until further analysis. For this study, 40 patients were selected on the basis of the availability of frozen tissue from which RNA of sufficient quality could be extracted. The clinicopathological characteristics of the patients were collected from the medical records, and the tumor stages were classified according to the American Joint Committee on Cancer TNM staging system. We evaluated the histopathological characteristics of the tumor specimens (i.e., histological grade [differentiation], vascular invasion, lymphatic invasion, and perineural invasion) by reviewing each slide stained with hematoxylin and eosin.

### Statistical analysis

The data obtained in the *in vitro* experiments are presented as mean ± standard deviation (SD). The mRNA expression levels of CDH1, SIP1, Snail, Twist, and Cox2 in the clinical samples are indicated as median values and ranges because of the skewed distribution of the data. Differences in the mRNA expression levels between paired samples (tumor vs. noncancerous) were assessed using the Wilcoxon signed rank-sum test. Correlations between the mRNA expression levels and clinicopathological factors were evaluated using the Mann-Whitney U-test or the Spearman rank correlation coefficient. Risk factors of lymph node metastasis were examined using Fisher’s exact test, the chi-square test, or the Mann-Whitney U-test for the univariate analysis, and a multiple logistic regression model with the stepwise selection method for the multivariate analysis. P-values less than 0.05 were considered statistically significant. All statistical analyses were performed using SPSS Ver. 16.0.

## Results

### Baseline mRNA expression of Cox-2, CDH-1, and its transcriptional repressors in HNSCC Cells

**Figure 1:**
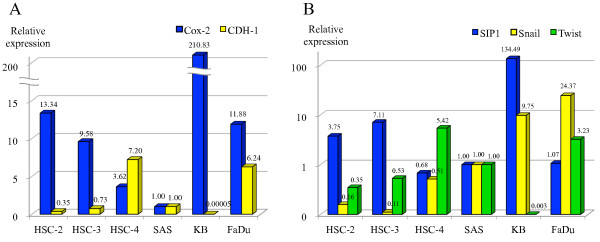
**Baseline mRNA expression of Cox-2, CDH-1 and its transcriptional repressors in HNSCC cells.** The mRNA expression levels of each gene in the HNSCC cell lines were assessed by quantitative real-time PCR. The relative expression levels were normalized by dividing each value by that of SAS as a calibrator for convenience. **A**: Cox-2 and CDH-1. **B**: SIP1, Snail, and Twist. While a trend toward an inverse correlation was found between Cox-2 and CDH-1 (r_s_ = −0.714, p = 0.055), SIP1 was shown to significantly correlate with Cox-2 (r_s_ = 0.771, p = 0.042) and to inversely correlate with CDH-1 (r_s_ = −0.886, p = 0.024) by Spearman rank correlation coefficient.

We used quantitative real-time PCR to evaluate the mRNA expression levels of Cox-2, E-cadherin transcripts (CDH-1) and its transcriptional repressors (SIP1, Snail, and Twist) in HNSCC cell lines. The relative expression levels of each gene were normalized by dividing each value by that of SAS cells as a calibrator for convenience. As shown in Figure 1A, a trend toward an inverse correlation was found between Cox-2 and CDH-1 by Spearman rank correlation coefficient (r_s_ = −0.714, p = 0.055). HT-1080 cells showed no CDH-1 expression as expected as the negative control for E-cadherin. Figure 1B displays the relative expression levels of the transcriptional repressors. Interestingly, the expression level of SIP1 was revealed to be significantly correlated with that of Cox-2 (r_s_ = 0.771, p = 0.042) and inversely correlated with that of CDH-1 (r_s_ = −0.886, p = 0.024), whereas those of Snail and Twist were shown to correlate with neither Cox-2 nor CDH-1.

Based on these baseline mRNA expression levels, we selected the following cells for the *in vitro* experiments: HSC-2 expressing a relatively high level of Cox-2 and a low level of CDH-1, and HSC-4 expressing a relatively low level of Cox-2 and a high level of CDH-1.

### Alterations in the mRNA expressions of CDH-1 and its transcriptional repressors by Cox-2 inhibition

**Figure 2:**
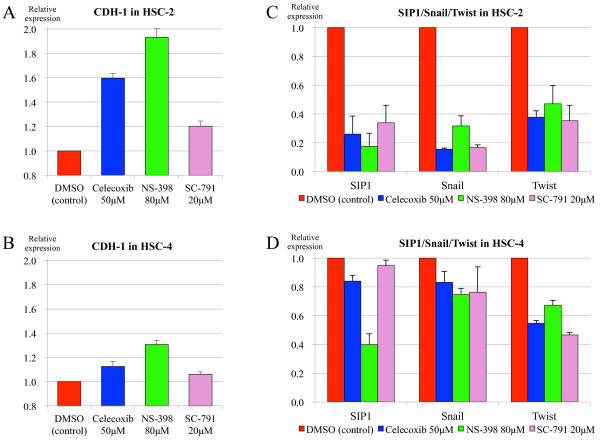
**Alterations in the mRNA expression of CDH-1 and its transcriptional repressors by Cox-2 inhibition.** The effect of Cox-2 inhibition on the mRNA expressions of CDH-1 and its transcriptional repressors (SIP1, Snail, and Twist) was examined by quantitative real-time PCR using three different selective Cox-2 inhibitors: celecoxib, NS-398, and SC-791. **A**: In HSC-2 cells, Cox-2 inhibition upregulated the CDH-1 expression compared to DMSO treatment as the control. **B**: In HSC-4 cells, Cox-2 inhibition resulted in relatively less upregulation of CDH-1 expression. **C**: In HSC-2 cells, all three transcriptional repressors were clearly downregulated by each of the Cox-2 inhibitors. **D**: In HSC-4 cells, Cox-2 inhibition led to relatively less downregulation of these transcriptional repressors.

We examined the effect of Cox-2 inhibition on the mRNA expressions of CDH-1 and its transcriptional repressors in the cell lines HSC-2 and HSC-4, using the three selective Cox-2 inhibitors celecoxib, NS-398, and SC-791. As regards the dose and exposure time of Cox-2 inhibitor, because we observed neither time-dependent nor dose-dependent manner in the regulation with each Cox-2 inhibitor in our preliminary experiments, the results were shown with the doses and exposure times considered to be optimal for each Cox-2 inhibitor and each purpose. In the HSC-2 cells, Cox-2 inhibition upregulated the CDH-1 expression compared to DMSO treatment as the control, increasing by 1.60-, 1.93-, and 1.20-fold with celecoxib, NS-398, and SC-791, respectively (Figure 2A). In contrast, Cox-2 inhibition in the HSC-4 cells resulted in relatively less upregulation of CDH-1 expression (Figure 2B). These results suggest that the extent of the effect of Cox-2 inhibition may vary depending on the cell type and presumably on the baseline expression levels of both CDH-1 and Cox-2 in each cell.

In line with these results, all three transcriptional repressors of E-cadherin were clearly downregulated in the HSC-2 cells by each of the Cox-2 inhibitors, with decreasing by 0.18–0.34-, 0.15–0.32-, and 0.35–0.47-fold in SIP1, Snail, and Twist, respectively (Figure 2C), whereas the Cox-2 inhibition in the HSC-4 cells led to relatively less downregulation of these transcriptional repressors (Figure 2D).

### Restoration of membranous E-cadherin expression by Cox-2 inhibition

**Figure 3:**
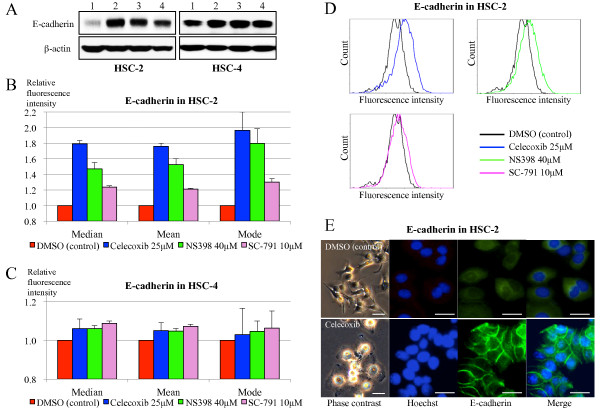
**Restoration of membranous E-cadherin expression by Cox-2 inhibition.** The alteration of E-cadherin protein expression following Cox-2 inhibition was evaluated using the selective Cox-2 inhibitors: celecoxib, NS-398, and SC-791. **A**: Western blot displayed that Cox-2 inhibition remarkably upregulated the protein expression of E-cadherin in HSC-2 cells compared to DMSO treatment as the control, whereas relatively less upregulation of E-cadherin was shown in HSC-4 cells. (Lane 1, DMSO; 2, Celecoxib 25 μM; 3, NS-398 40 μM; 4, SC-791 10 μM) **B**: E-cadherin expression on the cell surface was analyzed by flowcytometry. In HSC-2 cells, Cox-2 inhibition elevated the membranous expression of E-cadherin compared to DMSO treatment as the control. **C**: Cox-2 inhibition in HSC-4 cells resulted in a slight increase of E-cadherin expression. **D**: Histograms of the membranous expression of E-cadherin in HSC-2 cells with or without Cox-2 inhibition. **E**: Phase contrast images and immunofluorescent E-cadherin staining of HSC-2 cells. Cox-2 inhibition with celecoxib resulted in the restoration of the epithelial morphology to a polygonal shape, and enhanced intercellular expression of E-cadherin. Scale bar: 20 μm.

The Cox-2 inhibition-induced upregulation of E-cadherin in the HNSCC cells at protein level was confirmed by Western blotting (Figure 3A). In accord with its mRNA expressions, E-cadherin expression in the HSC-2 cells was noticeably enhanced by each of the Cox-2 inhibitors compared to DMSO treatment, whereas relatively less upregulation of E-cadherin expression was shown in the HSC-4 cells.

Because the function of E-cadherin in intercellular adhesion is maintained through the membranous localization of this molecule, we also evaluated the alteration of its protein expression on the cell surface using a flowcytometer. In line with aforementioned results, Cox-2 inhibition elevated the cell surface expression of E-cadherin compared to DMSO treatment in the HSC-2 cells, increasing by more than 1.76-, 1.47-, and 1.21-fold with celecoxib, NS-398, and SC-791, respectively (Figure 3B and D), whereas Cox-2 inhibition in the HSC-4 cells resulted in a slight increase of E-cadherin expression by less than 1.10-fold with any of the inhibitors (Figure 3C).

The cellular morphology and the localization of E-cadherin expression in the HSC-2 cells were further evaluated by a phase contrast microscope and immunofluorescent staining, respectively. As shown in Figure 3E, Cox-2 inhibition with celecoxib resulted in the restoration of the epithelial morphology to a polygonal shape, and enhanced intercellular expression of E-cadherin compared to DMSO treatment.

### Comparison of the mRNA expressions of Cox-2 and CDH-1 between TSCC and corresponding noncancerous tissues

From among the 40 patients with TSCC whose fresh-frozen tumor specimens were available for the present study, corresponding noncancerous mucosal tissues were also collected in 20 patients. In these paired samples, as shown in Table 1, the Wilcoxon signed rank-sum test revealed that the mRNA expression level of Cox-2 was significantly higher in the TSCC tissues than in the adjacent noncancerous mucosal tissues (median values, 5.865 vs. 3.707, p = 0.018). In contrast, the CDH-1 mRNA expression level was significantly lower in the TSCC tissue than in the noncancerous mucosal tissue (median values, 11.249 vs. 17.639, p = 0.024). However, no significant inverse correlation between Cox-2 and CDH-1 expression was observed in these samples, or only in 40 TSCC tissues.

**Table 1:** Comparison of gene expression levels between TSCC and corresponding noncancerous tissues

		**TSCC tissue (n = 20)**	**Noncancerous tissue (n = 20)**	**p value**^**a**^
Cox-2	median	5.865	3.707	0.018*
	(range)	(0.427 - 52.766)	(0.394 - 24.626)	
CDH-1	median	11.249	17.639	0.024*
	(range)	(0.048 - 24.494)	(2.321 - 36.348)	

^a^Wilcoxon signed rank sum test.

*Statistically significant.

TSCC = tongue squamous cell carcinoma.

### Correlations between the mRNA expression levels of each gene and clinicopathological factors

We evaluated the correlations between the mRNA expression levels of each gene in the TSCC tissues and the clinicopathological factors of the 40 patients with TSCC, as shown in Table 2. Higher Cox-2 mRNA expression was significantly correlated with lymph node metastasis (p = 0.037), while lower CDH-1 expression was correlated with both advanced T-classification (p = 0.041) and lymph node metastasis (p = 0.020). Although the mRNA expressions of SIP1, Snail, and Twist were associated with neither lymph node metastasis nor T-classification, higher expression of each of these three genes was significantly correlated with the histological grade (p = 0.004, 0.021, and 0.019, respectively). Higher expressions of SIP1 and Twist were also correlated with perineural invasion (p = 0.016 and 0.008, respectively). None of the genes examined were associated with other clinicopathological factors, including age, gender, vascular invasion, and lymphatic invasion.

**Table 2:** Correlation between gene expression levels and clinicopathological factors

**Variable**		**n**		**Cox-2**	**p value**	**SIP1**	**p value**	**Snail**	**p value**	**Twist**	**p value**	**CDH-1**	**p value**
Age^a^	< 60	25	median	3.964	0.583	3.191	0.773	1.071	0.273	12.469	0.119	13.681	0.878
			(range)	(0.640 - 61.171)		(0.035 - 17.376)		(0.020 - 6.229)		(0.000 - 64.312)		(0.100 - 45.381)	
	≧ 60	15	median	4.443		2.926		0.936		6.947		13.881	
			(range)	(0.427 - 52.766)		(0.059 - 9.482)		(0.099 - 2.361)		(0.936 - 20.548)		(0.841 - 24.494)	
Gender^a^	Male	35	median	4.037	0.817	3.200	0.247	0.986	0.611	9.794	0.746	12.670	0.379
			(range)	(0.427 - 61.171)		(0.035 - 17.376)		(0.020 - 6.229)		(0.000 - 64.312)		(0.100 - 45.381)	
	Female	5	median	4.331		1.454		1.191		9.102		19.520	
			(range)	(3.223 - 6.581)		(0.677 - 7.218)		(0.562 - 2.361)		(5.989 - 12.900)		(5.367 - 23.448)	
T classification^b^	1	2	coefficient	rs = -0.264	0.114	rs = 0.089	0.583	rs = -0.017	0.919	rs = 0.223	0.170	rs = -0.327	0.041*
	2	10											
	3	22											
	4	6											
LN metastasis^a^	N (-)	15	median	2.399	0.037*	2.926	0.964	0.983	0.800	6.947	0.226	18.801	0.020*
			(range)	(0.427 - 6.092)		(0.059 - 11.250)		(0.193 - 5.137)		(0.000 - 42.360)		(0.841 - 45.381)	
	N (+)	25	median	4.443		3.602		1.094		12.037		10.688	
			(range)	(1.379 - 61.171)		(0.035 - 17.376)		(0.020 - 6.229)		(0.936 - 64.312)		(0.100 - 23.697)	
Histological grade^b^	I	21	coefficient	rs = 0.155	0.338	rs = 0.462	0.004*	rs = 0.374	0.021*	rs = 0.381	0.019*	rs = -0.026	0.873
	II	12											
	III	7											
Vascular invasion^a^	Negative	32	median	3.478	0.133	3.393	0.360	1.006	0.608	9.369	0.913	14.999	0.085
			(range)	(0.640 - 61.171)		(0.035 - 17.376)		(0.020 - 5.538)		(0.000 - 64.312)		(0.100 - 45.381)	
	Positive	8	median	10.759		2.250		1.264		9.794		7.799	
			(range)	(0.427 - 43.355)		(0.059 - 6.356)		(0.193 - 6.229)		(1.246 - 29.053)		(0.841 - 23.697)	
Lymphatic invasion^a^	Negative	22	median	4.037	0.800	3.939	0.195	0.936	0.554	9.027	0.554	15.966	0.192
			(range)	(0.640 - 61.171)		(0.035 - 11.250)		(0 020 - 5.137)		(0.000 - 64.312)		(1.373 - 38.234)	
	Positive	18	median	4.733		2.155		1.104		10.915		10.694	
			(range)	(0.427 - 60.921)		(0.059 - 17.376)		(0.086 - 6.229)		(0.936 - 31.933)		(0.100 - 45.381)	
Perineural invasion^a^	Negative	30	median	4.128	0.841	2.212	0.016*	1.006	0.286	7.720	0.008*	14.891	0.617
			(range)	(0.427 - 61.171)		(0.035 - 11.250)		(0.020 - 5.137)		(0.000 0 64.312)		(0.100 - 38.234)	
	Positive	10	median	5.247		6.345		1.114		13.886		11.907	
			(range)	(0.640 - 60.921)		(2.250 - 17.376)		(0.458 - 6.229)		(9.027 - 31.933)		(2.089 - 45.381)	

^a^Mann-Whithey U test, ^b^Spearman rank correlation coefficient.

*Statistically significant.

LN = lymph node, rs = correlation coefficient.

### Univariate and multivariate analyses of risk factors affecting lymph node metastasis

To determine the risk factors predictive of lymph node metastasis, we further examined the correlation of lymph node metastasis with other clinicopathological factors. As shown in Table 3, advanced T-classification was significantly correlated with lymph node metastasis (p = 0.036). All other clinicopathological factors showed no significant correlation with lymph node metastasis, although lymphatic invasion tended to be associated with lymph node metastasis (p = 0.069).

**Table 3:** Univariate analysis of clinicopathological factors predictive of lymph node metastasis

**Variable**		**n**	**LN metastasis (-) n = 15**		**LN metastasis (+) n = 25**		**p value**
Age^a^	< 60	25	9	(36.0%)	16	(64.0%)	0.724
	≧ 60	15	6	(40.0%)	9	(60.0%)	
Gender^a^	Male	35	15	(42.9%)	20	(57.1%)	0.081
	Female	5	0	(0.0%)	5	(100.0%)	
T classification^b^	1	2	1	(50.0%)	1	(50.0%)	0.036*
	2	10	7	(70.0%)	3	(30.0%)	
	3	22	4	(18.2%)	18	(81.8%)	
	4	6	3	(50.0%)	3	(50.0%)	
Histological grade^b^	I	21	7	(33.3%)	14	(66.7%)	0.551
	II	12	6	(50.0%)	6	(50.0%)	
	III	7	2	(28.6%)	5	(71.4%)	
Vascular invasion^a^	Negative	32	13	(40.6%)	19	(59.4%)	0.350
	Positive	8	2	(25.0%)	6	(75.0%)	
Lymphatic invasion^a^	Negative	22	11	(50.0%)	11	(50.0%)	0.069
	Positive	18	4	(22.2%)	14	(77.8%)	
Perineural invasion^a^	Negative	30	13	(43.3%)	17	(56.7%)	0.174
	Positive	10	2	(20.0%)	8	(80.0%)	

^a^Fisher’s exact test, ^b^Chi-square test.

*Statistically significant.

LN = lymph node.

We used a multiple logistic regression model to further analyze the variables that were significantly correlated with lymph node metastasis in the aforementioned univariate analyses. As shown in Table 4, lower CDH-1 mRNA expression alone, and not Cox-2 mRNA expression or T-classification, was found to be the independent risk factor affecting lymph node metastasis in this series (odds ratio = 0.905, p = 0.041).

**Table 4:** Multivariate analysis of factors predictive of lymph node metastasis

**Variable**	**Odds ratio**	**95% confidence interval**	**p value**^**a**^
T-classification	1.119	0.418 - 2.993	0.823
Cox-2	1.011	0.965 - 1.060	0.648
CDH-1	0.905	0.822 - 0.996	0.041*

^a^Multiple logistic regression model.

*Statistically significant.

## Discussion

Our *in vitro* results revealed that, in HNSCC cells, the selective Cox-2 inhibitors led to the suppression of the EMT by restoring the expression of E-cadherin through the downregulation of its transcriptional repressors. Moreover, the extent of the effect of Cox-2 inhibition was shown to depend on the baseline expression levels of both E-cadherin and Cox-2 in each cell; i.e., tumor cells expressing lower E-cadherin and higher Cox-2 are expected to be more sensitive to Cox-2 inhibition in terms of the restoration of E-cadherin expression. Such a finding is consistent with a previous study of bladder cancer cells using another Cox-2 inhibitor, etodolac. In that study, etodolac upregulated E-cadherin expression only in T24 cells, which express the highest level of Cox-2 and the lowest level of E-cadherin; it did not do so in 5637 cells or K47 cells, which express a lower level of Cox-2 and a higher level of E-cadherin [42]. Interestingly, using the same three bladder cancer cell lines and three different Cox-2 inhibitors (etodolac, celecoxib, and NS-398), Adhim et al. found that E-cadherin mRNA was enhanced in all three cell lines by at least two Cox-2 inhibitors in each cell line, although the fold of increase remained the highest in T24 cells [43]. These and our results suggests that the suitability of Cox-2 inhibitor application could be assessed by predicting its anti-cancer effects in advance based on the baseline expression level of Cox-2 and certain of its downstream effector molecules.

Aside from the use of Cox-2 inhibitors, the Cox-2-dependent regulation of E-cadherin expression in HNSCC cells was demonstrated in a study using KB cells transfected with Cox-2 cDNA and gene silencing with Cox-2 siRNA, although the specific signaling pathway between Cox-2 and E-cadherin was not referred to [45]. In HNSCC cells, St. John et al. elucidated that proinflammatory cytokine IL-1β induces downregulation of E-cadherin through the Cox-2/Snail pathway, which is blocked by the selective Cox-2 inhibition using celecoxib or Cox-2 small hairpin RNA [44]. Those findings also corroborate our results regarding the Cox-2 inhibition-induced restoration of E-cadherin expression in HNSCC.

Regarding the direct mechanisms underlying the downregulation of E-cadherin, it has been suggested that transcriptional repression and promoter hypermethylation are primarily responsible in sporadic carcinoma, whereas other mechanisms such as genomic deletion and loss of heterozygosity associated with germline mutation are observed in hereditary carcinoma [6-8]. According to the study that examined CpG island methylation around the promoter region of CDH-1 in HNSCC cell lines by methylation-specific PCR, the methylation was partially found in the HSC-2 cells, but not in the HSC-4 cells [46], which may also accounts for the low base-line expression of E-cadherin in the HSC-2 cells.

In our present *in vitro* study, the mRNA expression level of SIP1, but not those of Snail or Twist, showed a significant inverse correlation with that of CDH-1, which is in agreement with previous findings in HNSCC, breast, and hepatocellular carcinoma cells [9,47-49]. We observed that the SIP1 expression was also significantly correlated with Cox-2, suggesting the possibility that SIP1 acts as a principal effector in the Cox-2-dependent regulation of E-cadherin expression in HNSCC. However, the Cox-2 inhibitors used in the present study led to the downregulation of not only SIP1 but also Snail and Twist comparably, indicating the similar importance of each transcriptional repressor in this pathway. In NSCLC cells, ZEB1 and Snail were found to be repressors responsible for the regulation of E-cadherin downstream of Cox-2/PGE_2_[37], whereas in bladder cancer cells Cox-2 inhibitors downregulated all of the E-cadherin repressors examined: Snail, Slug, Twist, and ZEB1 [43]. Aside from the implication of Cox-2, in breast cancer cells, receptor activator of NF-κB ligand (RANKL) was revealed to downregulate the E-cadherin expression by activating the NF-κB pathway and enhancing Snail and Twist expression [50]. In HNSCC cells, inhibition of Akt activity was shown to decrease NF-κB signaling, thereby downregulate the expression of Snail and Twist, but not SIP-1, to induce the mesenchymal-to-epithelial reverting transition [51]. Although we did not evaluate the expressions of ZEB1 and Slug, our results verified these reports in terms of the direct role of transcriptional repressors in the Cox-2-dependent regulation of E-cadherin.

In addition to the suppression of the EMT, some other anti-cancer effects of Cox-2 inhibitors in HNSCC have been reported, which include the inhibition of VEGF-A expression by celecoxib [15], the suppression of invasiveness by NS-398 [52,53] and celecoxib [54], the inhibition of proliferation by celecoxib, NS-398, nimesulide, and meloxicam [54,55], and the induction of apoptosis by celecoxib [55]. Since a close relationship is likely between the EMT and enhanced cell migration, the Cox-2 inhibitor-induced suppression of the EMT may also contribute to the attenuation of the invasiveness of cancer cells. Considering the multifaceted function of Cox-2 itself, a variety of mechanisms are thought to be involved in the anti-cancer effects of selective Cox-2 inhibitors, and these mechanisms are presumed to exert their effects cooperatively.

In the clinical samples that we examined, compared to adjacent noncancerous mucosal tissue, the mRNA expression level of CDH-1 was significantly lower in the TSCC tissue as expected, although functional E-cadherin is supposed to be assessed by its membranous expression. In addition, we found that the mRNA expression level of Cox-2 was significantly higher in the TSCC tissue, which is consistent with the previous studies including those that examined HNSCC [14,15]. As for a possible inverse correlation between Cox-2 and E-cadherin expressions, we found a trend toward an inverse correlation in the HNSCC cell lines examined, whereas no correlation was observed in the clinical samples of TSCC. Inconsistent statistical results have been reported even in immunohistochemical evaluations of cancers other than HNSCC: although a significant inverse correlation between Cox-2 and E-cadherin expressions was seen in bladder cancer [41], no correlation between them was revealed in gastric cancer [40], the latter of which is in agreement with our result assessed by quantitative real-time PCR. Such discrepancies could be attributed not only to differences in the sites of cancer origin and sample size, but also to differences in the studies’ evaluation methods and statistical methods. Aside from these statistical analyses, an inverse expression pattern between Cox-2 and E-cadherin in each of individual cases was seen by immunohistochemical observation in NSCLC and colon cancer [37,56]. Considering tissue heterogeneity in terms of the localized expression of particular molecules along with the above-mentioned immunohistochemical observation, we speculate that the extent of the upregulation of Cox-2 and its possible downregulation of E-cadherin may depend on microscopically specific sites such as the invasive front or the inside of cancer nests, which would not necessarily be reflected in any statistical analysis or in homogenized samples at all.

Regarding the correlations with clinical parameters in TSCC, while univariate analysis demonstrated that the mRNA expressions of both higher Cox-2 and lower CDH-1 were significantly correlated with lymph node metastasis, the multivariate analysis revealed that a lower CDH-1 mRNA expression level was the only independent predictor of lymph node metastasis in this cohort. These results suggest that the induction of the EMT, regardless of dependency on its various upstream pathways, is closely implicated in the development of lymphogeneous metastasis. However, the predictive reliability of a lower CDH-1 mRNA expression level should be further validated using much larger independent cohorts. The result regarding Cox-2, even though it was confined to the univariate analysis, is in accord with the preceding immunohistochemical studies of HNSCC, although those were also missing multivariate analysis [15,16]. Considering its role in the regulation of E-cadherin expression, Cox-2 is thought to indirectly contribute to lymph node metastasis, at least in part through the induction of the EMT. On the other hand, our result regarding CDH-1 is consistent with the previous immunohistochemical studies of oral SCC that reported a significant correlation between reduced E-cadherin expression and lymph node metastasis [57-60], but not with others that showed no correlation between them [61-63], although all of those studies lacked multivariate analysis. These contradictory results seemed to be attributable to the quite variable criteria used to evaluate the extent of immunostaining intensity, which inevitably seems prone to subjective judgment. In addition, since each tumor specimen consists of heterogeneous cancer cell populations that show different behaviors, staining scores could vary depending on the tumor portion selected for examination. To overcome such uncertainties accompanying immunohistochemical evaluation, instead we quantified mRNA expression levels in homogenates from whole frozen blocks of tumor samples. However, those data must still be interpreted cautiously because the differences in expression levels according to microscopically distinct sites and cellular localization cannot be considered, and it is thus possible that certain correlations would be missed.

Practically, if clinical N0 (cN0) patients with occult lymph node metastasis can be discriminated accurately from other cN0 patients, we could apply neck dissection exclusively for those selected patients in advance of the inevitable development of delayed neck metastasis. Therefore, from a clinical point of view, the prediction of lymph node metastasis is genuinely meaningful in cN0 cases. Among the reliable studies conducted to identify predictive markers of delayed or occult neck metastasis within clinical stage I/II (cT1-2 N0) oral squamous cell carcinoma by a multivariate analysis, tumor thickness or depth has been most accepted as an independent histopathological parameter [64]. However, the downregulation of E-cadherin was recently revealed by us and others to be one of the independent molecular predictors of delayed neck metastasis even in such a limited patient population [9,65,66]. These previous and present results suggest that the restoration of E-cadherin expression by inhibiting any of the upstream signals promoting the EMT may prevent the initiation and progression of lymph node metastasis of HNSCC. Further investigations are indispensable to establish the optimal standard to evaluate the risk of metastasis using molecular markers related to the EMT.

In conclusion, our findings suggest that the downregulation of CDH-1 resulting from the induction of the EMT is closely involved in lymph node metastasis in HNSCC. The expression profiles of EMT-related molecular makers in primary tumors are thought to be informative to predict the clinicopathological behavior of HNSCC. In addition, the appropriately selective administration of selective Cox-2 inhibitors may lead to an anti-metastatic effect as suppression of the EMT by restoring E-cadherin expression through the downregulation of its transcriptional repressors, cooperatively with various other mechanisms.

## Competing interests

There are no financial or other relationships that may lead to a conflict of interests.

## Authors’ contributions

RF and YI (contributed equally) conceived of and designed the study, conducted the experiments, performed the data analysis, and drafted the manuscript. KS and NS carried out the experiments. KS, SS, NH, and KOt participated in the design of the study and conducted the experiments. YS and YW supported the experiments and the data analysis. KK provided and reviewed the histopathological diagnosis of clinical specimens. HO, TT, and MF participated in the design of the study and the data analysis. KOg provided general support to conception of the study. All authors read and approved the final manuscript.
